# Global Traction Battery Cathode Material Industrial Chain Trade Analysis: A Multilayer Modeling Approach

**DOI:** 10.3390/e26110895

**Published:** 2024-10-23

**Authors:** Peng Peng, Yang Xu, Li Yu, Xiaowei Xie

**Affiliations:** 1 State Key Laboratory of Resources and Environmental Information System, Institute of Geographic Sciences and Natural Resources Research, Chinese Academy of Sciences, Beijing 100101, China; pengp@lreis.ac.cn (P.P.); xuy@lreis.ac.cn (Y.X.); xiexw@lreis.ac.cn (X.X.); 2 University of Chinese Academy of Sciences, Beijing 100049, China; 3 Center for Strategic Research on Frontier and Interdisciplinary Engineering Science and Technology, Beijing Institute of Technology, Beijing 100081, China

**Keywords:** global traction battery trade, multilayer network, electric vehicle, lithium-ion battery

## Abstract

The fast expansion of the electric vehicle market has led to a significant increase in the demand for traction batteries, an essential element in these vehicles that provide the opportunity to achieve low-carbon and environmentally friendly growth and carbon neutrality. By analyzing the network structure and key trading countries from 2000 to 2021, this research uses multilayer network theory to explore the trade patterns and the evolution of the global cathode material industrial chain of traction batteries. Our findings indicate the following: (1) The industrial networks display multi-core trading country characteristics. Trade connections among the top 20 countries, which account for more than 80% of the global trade volume, have strengthened. (2) Over time, the geographic center of trade has shifted from being primarily focused in Europe, North America, and East Asia to embracing the entire world, including regions such as Africa, South America, and Oceania. (3) In 2021, Australia overtook Japan as the main exporter, which held the top position in 2000. Similarly, China surpassed the United States, which was the top importer in 2000. (4) Changes in global trade relationships have affected the trading habits of the top-ranked countries.

## 1. Introduction

Owing to scientific and technological progress, together with the pursuit of low-carbon and eco-friendly growth and carbon neutrality goals, the global automobile industry has witnessed a significant rise in the use and promotion of electric vehicles. The global sales of electric vehicles exceeded 10 million for the first time in 2022, reaching a total of 10.824 million [1]. Traction batteries serve as the central element of electric vehicles, and the demand for these batteries has witnessed a substantial increase as a result of the rapid expansion of the electric vehicle market. After twenty years of development, the traction battery industry has developed a specialized chain structure with clear division of labor, covering activities from mineral extraction to battery manufacturing. Consequently, complex trade networks have emerged among different countries. A worthwhile field of study to investigate is the worldwide traction battery trade patterns and their evolution over time. This will facilitate strategic planning and policy-making in the context of the global trade security of traction batteries.

Recently, scholars have carried out a range of studies primarily using trade data, focusing on prominent trading countries, significant trade patterns, and the potential trade risks associated [2,3,4] with the upstream mining sector for traction batteries and downstream Li-ion battery products. This includes examining the impact of specific cobalt mines [5], as well as analyzing the complex trade network relationships involving key minerals in the upstream industrial chain [6]. These studies serve as valuable theoretical references for understanding the trade relations within the global traction battery downstream industrial chain. They also effectively highlight the risks that exist in these trade relationships [7]. The traction battery industrial chain is a complex system that involves various stages. It includes the extraction of mineral raw materials such as nickel, cobalt, and lithium in the upstream, and in the downstream, it encompasses the production of lithium battery products. Additionally, precursors act as a crucial intermediary products that connect the upstream resources and downstream materials in the battery industrial chain [8]. The midstream of the industrial chain consists of cathode materials, anode materials, diaphragms, electrolytes, and structural components, all of which play a significant role in the traction battery industrial chain [9]. The current literature fails to consider the entire industrial chain in order to establish global trade links for traction battery trade.

The cathode material constitutes almost 40–50% of the total cost of a traction battery, making it the primary factor in determining the battery’s cost [10]. Within cathode material production, the cost of the basic raw cathode materials constitutes around 90% of the total cost. Hence, this paper specifically examines the trade relations involving cathode materials used in traction battery industrial chains. Based on the division of the industrial chain, the traction battery industry can be categorized into the upstream, precursor, midstream, and downstream sectors [11]. The upstream involves the extraction and processing of raw materials that are essential for manufacturing batteries. Key materials include lithium, cobalt, nickel, manganese, graphite, and aluminum, among others. Activities in this segment include mining, refining, and the production of battery-grade chemicals and materials [12]. The midstream refers to the manufacturing and assembly of battery cells, modules, and packs. It involves the conversion of raw materials and components into finished battery products ready for integration into EVs or energy storage systems [13]. The downstream pertains to the application and recycling of used batteries. It includes the integration of battery packs into electric vehicles, energy storage solutions, and other applications like consumer electronics. Additionally, it encompasses the end-of-life management of batteries, including recycling processes to recover valuable materials and ensure environmental sustainability [14]. Traditional industry classifications do not always explicitly list precursor materials as a separate category; they are an integral part of the supply chain, bridging the gap between upstream raw materials and midstream battery manufacturing. These are specialized chemicals or compounds used directly in the synthesis of cathode and anode materials for lithium-ion batteries. Examples include lithium carbonate for cathodes and synthetic graphite for anodes. The production and supply of these precursors involve sophisticated chemical engineering processes and play a crucial role in determining the overall quality, cost, and environmental impact of batteries [15].

This paper utilizes United Nations Commodity Trade (UNComtrade) data from 2000 to 2021 to create a comprehensive trade network for the traction battery industrial chain. This network includes multiple layers representing the upstream, precursor, midstream, and downstream sectors. The objective is to analyze the evolution of trade relations using a multilayer complex network approach and identify trends in the trade relations of key countries. Countries from single industrial chains (e.g., upstream, precursor, midstream, and downstream sectors) tend to trade with other countries and have distinct trade patterns, resulting in the establishment of some specific single-layer subnetworks. Among these, some critical countries can play a variety of roles when considering various single-layer subnetworks. As a result, the entire traction battery industrial chain can be represented as a multilayer network comprising complimentary single-layer subnetworks. These essential countries serve critical roles in the overall coherence of the trade network structure, connecting various forms of trade interactions. We define them as versatile countries using the concept of a versatile node proposed in earlier research [16].

The single-layer network model is commonly used in network analysis for traction battery trade [5,17]. The UNComtrade classifies 11 categories (layers) of commodities for the traction battery industrial chain using a six-digit code. Using single-layer network models to investigate 11 layers of the traction battery industrial chain independently can be overwhelming in terms of the amount of detail. Multilayer network modeling allows for the representation of complex systems with multiple interconnected layers, capturing hierarchical relationships and facilitating deeper insights into the underlying structures and dynamics [18]. Therefore, we propose a multilayer network model, which divides each industrial chain’s commodities into upstream, precursor, midstream, and downstream layers. A multilayer modeling approach should retain the specific properties of different industrial chains.

This paper utilizes multilayer modeling to understand the diverse roles and trade interactions that the global traction battery industrial chain trade network engages in. It also aims to identify versatile countries and emerging properties in this trade network using different industrial chains. The primary research contribution of this study is twofold. On one hand, the industrial chain of traction batteries is established, encompassing the upstream, precursor, midstream, and downstream sectors. The application of a multilayer complex network methodology further enriches this, enabling an exhaustive analysis of the interrelations between the various industrial chains while maintaining their unique properties. On the other hand, this study examines the trade patterns and the evolution of the global traction battery industrial chain trade network empirically. By synthesizing the UNComtrade trade data for traction battery industrial chains from 2000 to 2021, it offers an in-depth exploration of trade network structure evolution and the trade dynamics amongst key participating countries, thereby providing valuable insights into the global traction battery market.

The structure of this paper is as follows: Section 2 provides an overview of the existing literature. Section 3 provides an explanation of the methodology and the data used. Section 4 presents the results and the related analysis. The final section encompasses our concluding findings and the implications for policy.

## 2. Literature Review

Accelerating the advancement of electric vehicles is a crucial way to achieve the United Nations’ Sustainable Development Goals. Many countries are focusing on bolstering the electric vehicle market in order to mitigate the adverse environmental and energy effects of the transportation industry [19]. There is expected to be a substantial increase in electric vehicle sales over the coming years, from 5 million units sold annually in 2015 to approximately 180 million units by 2045 [20]. As the core component of an electric vehicle fleet, the entirety of the traction battery industrial chain has also experienced dramatic growth with the increasingly strong demand for electric vehicles. The traction battery industrial chain encompasses multiple interconnected stages and a diverse range of materials, spanning from crucial upstream mineral resources to downstream lithium battery products. Therefore, ensuring the stability and security of the supply chain is of utmost importance for the seamless development of the whole traction battery industrial chain. Scholars have analyzed the stock distribution patterns of important countries and their trade connections in relation to lithium and cobalt ores, which are crucial for traction batteries [21]. The objective is to gain a comprehensive understanding of the reserves and trade dynamics of these countries involved in raw material production. For example, China is an important country in the lithium trade and consumes almost 50% of the lithium used worldwide. Western Sichuan, northern Xinjiang, and the Qinghai–Tibet Plateau possess 60.5% of China’s solid ore lithium and 86.8% of its liquid brine lithium. Transportation bottlenecks and high extraction costs limit domestic supply, making extraction difficult. To meet its lithium demands, China mostly imports lithium from Australia and Chile [22]. The imperative need to enhance global collaboration in order to ensure the availability of lithium supplies and other essential minerals has been extensively demonstrated [23]. Hence, a complex trade network is formed based on demand and supply of critical resources between countries. Nevertheless, these intricate relationships are vulnerable to perpetual fluctuations caused by multiple factors. Many scholars have discovered that several factors, such as political events, natural disasters, and environmental regulations [24,25], have a significant impact on the global supply network. Additionally, there are supply risks associated with crucial materials across these supply chains. These researchers raised attention to the fact that the world’s shift to renewable energy sources has introduced additional difficulties, such as the effects of price fluctuations and geopolitical vulnerabilities on the prices of essential metals used in global trade [26,27]. The above-mentioned studies identified the key countries involved in these industrial chains and the primary factors that influence the risks related to the traction battery industrial chain. They provide valuable insights into understanding the trade characteristics of the traction battery industrial chain. However, there is also a clear relationship of association and mutual impact among the countries involved in traction battery trading, resulting in a complex network. However, the aforementioned research was not conducted from a global viewpoint.

For a system-level analysis of international connections, complex network theory is an effective tool. Single-layer network analysis refers to the study of networks that consist of a single layer of nodes and edges. These networks can represent various types of relationships between entities, such as friendships in social networks or interactions between genes in a biological network [28,29]. Scholars investigate the trade relationship between countries in the global traction battery industrial chain using complex network modeling [3,17]. Key trade countries are essential for maintaining the stability of the global industrial chains. Scholars have efficiently identified important countries in many industrial chains by utilizing complex network centrality indices, such as degree value and betweenness centrality. For example, Sun et al. developed a comprehensive trade-linked material flow analysis model to examine the flow of cobalt throughout its anthropogenic life cycle and across different countries from 1995 to 2015. The findings suggest that the Democratic Republic of the Congo, the United States, China, and Japan hold significant influence in the cobalt trading network that spans across the world [5]. Scholars have also examined the localized aggregation of relationships among countries engaged in power cell trade using the community detection method, in order to further investigate the unique trade characteristics of specific regions [17]. Chen et al. analyzed the evolution of lithium trade communities from 1990 to 2017. Their findings indicate that the trade of lithium carbonate can be broadly categorized into three main trading communities: Asia, North America, and Europe. Similarly, the trade of lithium hydroxide can also be roughly divided into three major trading communities: Asia–North America, Europe centered around the United Kingdom, and Europe centered around France [3]. In addition to analyzing the international trade structure, the supply risk within the trade structure is also a very hot topic [7,30]. Hu et al. introduced the trade network risk transmission (TNRT) model to analyze hidden systemic risks in the worldwide EV-LIB trade between 2012 and 2019, specifically focusing on two supply shortfall scenarios. Their findings indicate that the worldwide EV-LIB trade network has a “robust-yet-fragile” nature, and they also expose hidden risks in the top EV-LIB exporter nodes and trade edges [30]. However, there exist strong correlations and reciprocal influences among various segments of industrial chains, ranging from upstream minerals to downstream traction battery products. For example, the suspension of imported lithium causes upstream providers to be unable to meet the needs of downstream sectors, while the interruption of exported lithium causes downstream industries to decrease their demand for raw materials, leading to a backlog in upstream production [31]. Hence, the utilization of single-layer complex networks fails to adequately uncover the inherent correlation characteristics between the trade relations of distinct industrial chains.

Theoretically, a multilayer network model incorporates numerous networks, or “layers”, into a single mathematical framework [18]. Multilayer network analysis, also known as multiplex network analysis, involves studying networks that consist of multiple layers or types of relationships between the same set of nodes. Each layer in a multilayer network represents a different type of interaction or relationship among the same entities. This approach allows for a more nuanced understanding of complex systems where nodes can engage in different types of interactions simultaneously [32,33,34]. It allows us to model various trade relations among the same group of countries, with each layer representing a distinct sort of trade commodity. Many theoretical and experimental studies have demonstrated that the analysis of multilayer networks has led to significant progress compared to approaches that only consider single-layer networks [16,35]. Hence, the use of the multilayer complex network approach offers solid research support for this paper’s analysis of the trade relationship within the entire industrial chain of traction batteries. In recent years, the multilayer network model has garnered significant interest from scholars [36,37,38]. Several network indices often employed in single-layer network analysis have been customized to be applicable in multilayer network modeling [32,33,39]. Thus far, multilayer modeling has been utilized in various domains, including the study of human brain networks [40], animal behavior networks [41], global trade networks [42], and transportation networks [35,43]. When it comes to multilayer modeling for traction battery studies, scholars have focused extensively on the essential mineral trade and the associated trading risks. Shao et al. investigated the multiplex cobalt-lithium trade network (CLTN) from 2010 to 2019, finding that CLTN continues to globalize and that cobalt and lithium trade are becoming increasingly interconnected. With the development of low-cobalt technology, the impact of the cobalt trade layer on the lithium trade layer is becoming increasingly apparent [6]. Hao et al. studied the dynamic process of risk transmission along the lithium industrial chain using multilayer risk transmission networks (MRTN) to estimate risk magnitude and pathway. Their findings show that the lithium trade network’s risk transmission involves “minority communication” and is “robust-yet-fragile”. Increasing trade links with multiple partners gives network countries more flexibility and risk management. However, hub countries may lose dominance [2].

## 3. Data and Methodology

### 3.1. Data

This study utilized data obtained from the UNComtrade Database (https://comtrade.un.org/). This database is a completely trustworthy and extensively utilized repository that encompasses all the international trade movements of exports and imports between countries and regions across the world. Peng et al. [44] found that data on exports are generally more dependable and are commonly used in this setting. We selected the trade value, measured in USD, as the unit of measurement for the network’s weight. UNComtrade provides comprehensive data on the import and export operations of nations, organized by product using the Harmonized Commodity Description and Coding System (HS). The HS codes and data descriptions are listed in Table 1.

### 3.2. Network Construction

**Figure 1:**
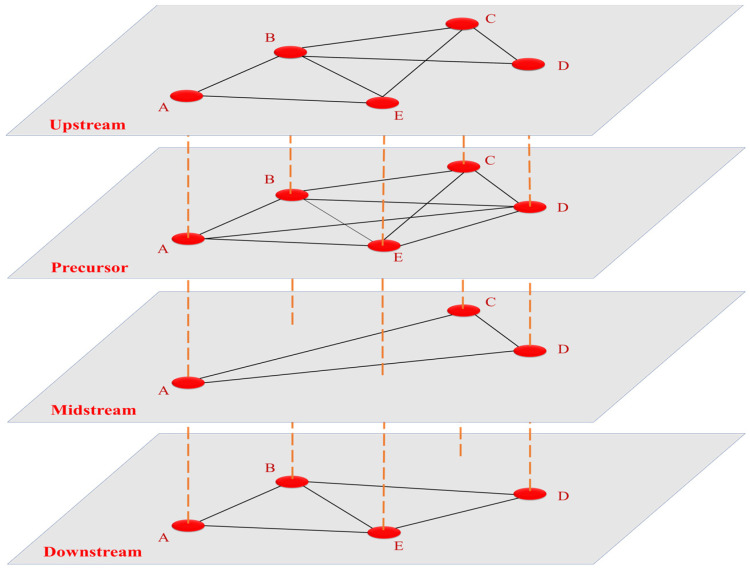
Schematic representation of the multilayer network.

We introduced a multilayer modeling approach for the exploration of the traction battery trade. The multilayer network depicted in Figure 1 consists of red nodes representing countries and black lines representing actual trade relationships between the countries. The orange dotted lines represent the connections between countries in each layer, indicating the varying roles played by a certain country node in successive layers, as it is related to different nodes and countries in other layers. For instance, node (country) A being in all four layers signifies its involvement in trade across all four industrial chains. Additionally, node (country) A plays a significant role as an exporter in the upstream, precursor, and midstream sectors, but not in the downstream sector. Similarly, node (country) B being in three layers, excluding the midstream layer, indicates its involvement in trade across three industrial chains, excluding the midstream layer.

More formally, let us define a multilayer graph $G=\left(G_{1},\;G_{2},\;\ldots ,\;G_{L}\right),$ where $G_{1}$, $G_{2}$, …, $G_{L}$ denote the layers and L denotes the number of layers (e.g., upstream, precursor, etc.). A given layer $G_{i}$ is defined as $G_{i}=\left(N_{i},E_{i},W_{i}\right)$, with $N_{i}$ being the set of nodes representing the countries involved in this layer, $E_{i}$ representing the connections between these countries, and $W_{i}$ representing the weight matrix associated with this layer (the weight between two countries expressed by the accumulated trade value in this relationship). For any country $n_{i}\;$, let $F\left(n_{i}\right)$ denotes the set of layers associated with this country—that is, $F\left(n_{i}\right)\subseteq G$ (which is a subset of G). We adopted a adjacency tensor $M_{\beta \tilde{\delta }}^{\propto \tilde{\gamma }}$ to represent the global traction battery trade network [34]. This adjacency tensor $M_{\beta \tilde{\delta }}^{\alpha \tilde{\gamma }}$ is a generalization of the concept of an adjacency matrix from graphs to hypergraphs, and it is formally defined as follows:

$$\begin{array}{l} M_{\beta \tilde{\delta }}^{\alpha \tilde{\gamma }}=\sum_{\tilde{h},\tilde{k}=1}^{L}C_{\beta }^{\alpha }\left(\tilde{h}\tilde{\;k}\right)E_{\tilde{\delta }}^{\tilde{\gamma }}\left(\tilde{h}\tilde{\;k}\right)\\ =\sum_{\tilde{h},\tilde{k}=1}^{L}\left[\sum_{i,\;j=1}^{N}w_{ij}\left(\tilde{h}\tilde{\;k}\right)E_{\beta }^{\alpha }\left(i\;j\right)\right]E_{\tilde{\delta }}^{\tilde{\gamma }}\left(\tilde{h}\tilde{\;k}\right)\\ =\sum_{\tilde{h},\tilde{k}=1}^{L}\sum_{i,\;j=1}^{N}w_{ij}\left(\tilde{h}\tilde{\;k}\right)\varepsilon_{\beta \tilde{\delta }}^{\alpha \tilde{\gamma }}\left(ij\tilde{h}\tilde{\;k}\right) \end{array}$$

where $w_{ij}\left(\tilde{h}\tilde{\;k}\right)$ represents the intensity of the real relationship (which may not be symmetric) between node $n_{i}$ in layer $\tilde{h}$ ($\tilde{h}=1,2,\ldots ,L$) and node $n_{j}$ in layer $\tilde{\;k}$ ($\tilde{k}=1,2,\ldots ,L$), and $\varepsilon_{\beta \tilde{\delta }}^{\alpha \tilde{\gamma }}\left(ij\tilde{h}\tilde{\;k}\right)\equiv e^{\alpha }\left(i\right)e_{\beta }\left(j\right)e^{\tilde{\gamma }}\left(\tilde{h}\right)e_{\tilde{\delta }}\left(\tilde{\;k}\right)$ indicates the fourth-order tensors consisting of the canonical basis in space $R^{N\times N\times L\times L}$. This study concentrates on interlayer connections, where each assigned node is linked to its corresponding node across multiple layers. It specifically examines the distinct responsibilities that each node (country) fulfills across various layers (different industrial chains).

### 3.3. Methodology

#### 3.3.1. Network Indices

In order to describe the underlying structure of a certain trade network, four indices were chosen: the shortest path (L), the clustering coefficient (C), Shannon entropy, and the assortativity coefficient.

The following equation represents the average topological distance between any two individual countries, which is used to compute the average shortest path length L of the network [45].

(1) $$\;L=\frac{1}{N\left(N-1\right)}\sum_{i\in V}\sum_{j\neq i\in V}d\left(i,j\right)$$

where $d\left(i,j\right)$ is the topologically shortest path length between countries *i* and *j* and *N* is the overall number of countries in the trade network.

*C*, the clustering coefficient [46], represents the degree to which a particular trade network’s countries are interconnected. The equation for *C* is as follows:

(2) $$C=\frac{1}{N}\sum_{v\in V}C_{v}$$

(3) $$C_{v}=\frac{\left|\left\{e_{ij}:i,j\in N_{v},e_{ij}\in E\right\}\right|}{\frac{k_{v}\left(k_{v}-1\right)}{2}}$$

where *N* represents the total number of countries in the network. $e_{ij}$ is the total number of edges in the network between all $k_{v}$ neighbors of country *v*, $C_{v}$ is the clustering coefficient of node *v*, $N_{v}$ is the set of all countries linked with country *v*, $k_{v}$ is the size of set $N_{v}$, and $\frac{k_{v}\left(k_{v}-1\right)}{2}$ is the maximum allowable number of edges between $k_{v}$ nodes.

Shannon entropy is a measure of the average degree of uncertainty or information related to the alternative states or potential outcomes of a random variable [47]. This metric quantifies the anticipated level of information required to characterize the condition of the variable, taking into account the probability distribution among all possible states.

(4) $$\varkappa =-\sum_{k=1}^{N-1}P\left(k\right)lnP\left(k\right)$$

where $P\left(k\right)$ represents the probability that the node has degree k. A high entropy value specifies a network that is more intricate and has a greater variety of connections, whereas a low entropy value suggests a network structure that is simpler and more regular.

The assortativity coefficient [47] can be utilized to examine the hierarchical distribution characteristics of countries in the traction battery commerce network.

(5) $$r=\frac{M^{-1}\sum_{i}{j_{i}k_{i}-\left(M^{-1}\sum_{i}\frac{1}{2\left(j_{i}+k_{i}\right)}\right)}^{2}}{M^{-1}\sum_{i}\frac{1}{2\left(j_{i}^{2}+k_{i}^{2}\right)}-{\left(M^{-1}\sum_{i}\frac{1}{2\left(j_{i}+k_{i}\right)}\right)}^{2}}$$

where $j_{i}$ and $k_{i}$ represent the degree values of the two ports connected by the *i*-th route (*i* = 1, 2, 3 … *M*, *M* is the total number of routes of the network). The magnitude of *r* lies within the range of −1 to 1. The network is assortative if r > 0, indicating that countries with high degree values are more likely to connect. No association between network structure is indicated by r = 0. A value of r < 0 indicates a disassortative network structure, where high-degree countries connect with low-degree countries.

#### 3.3.2. Versatile Countries Mining in Multilayer Network

Instead of relying on in- and out-degree centrality indices, we employed algorithms linked to the Hyperlink-Induced Topic Search (HITS) centrality metrics to illuminate network typology [48]. This approach describes each node, hub, and authority using two unique properties. HITS centrality metrics serve as a method for assessing the significance of web pages within a network. HITS has two distinct properties: authority scores assess a page’s importance based on the quantity and quality of incoming links from other high-authority pages. A higher authority score suggests that the page is well known and may provide useful information. The hub score measures a page’s influence by assessing the number and quality of outbound links to other high-hub pages. A higher hub score suggests that the page can spread its impact to other relevant pages. These scores are calculated iteratively until convergence, which occurs when the scores for each page stabilize. The final authority and hub scores provide an overall assessment of each page’s value inside the network [48]. In our study, hubs represent strong exporters in this structure, and authority represents strong importers. Under HITs, the topology of the network is similar to a bipartite topology, where the primary authorities are the nodes that receive a high volume of trade from important hubs. Consequently, nodes with a high hub centrality connect nodes with a high authority centrality, and very influential hubs direct their trade to equally influential authorities. Such a mechanism is characterized by the following two related equations:

(6) $${\left(WW^{†}\right)}_{j}^{i}v_{i}=\lambda_{1}v_{j}$$

(7) $${\left(W^{†}W\right)}_{j}^{i}z_{i}=\lambda_{1}z_{j}$$

where $W^{†}$ stands for the transposition of the adjacency matrix W, $\lambda_{1}$ is the leading eigenvalue, and $v_{i}$ and $z_{i}$ are the hub and authority scores, respectively.

The hub and authority equations are

(8) $${\left(MM^{†}\right)}_{j\beta }^{i\alpha }\Gamma_{i\alpha }=\lambda_{1}\Gamma_{j\beta }$$

(9) $${\left(MM^{†}\right)}_{j\beta }^{i\alpha }\Upsilon_{i\alpha }=\lambda_{1}\Upsilon_{j\beta }$$

where $\Gamma_{i\alpha }$ represents hub centrality, while $\Upsilon_{i\alpha }$ represents authority centrality. To obtain scores corresponding to each node, regardless of the layer, the hub and haven tensors should be contracted with the 1-vector. This can be represented as $\gamma_{i}=\Gamma_{i\alpha }u^{\alpha }$ and $v_{i}=\Upsilon_{i\alpha }u^{\alpha }$, respectively. In an undirected linked multilayer network, the hub and authority scores are identical and equivalent to the associated eigenvector centrality.

## 4. Results

### 4.1. Growing Trend in the Global Traction Battery Trade

**Figure 2:**
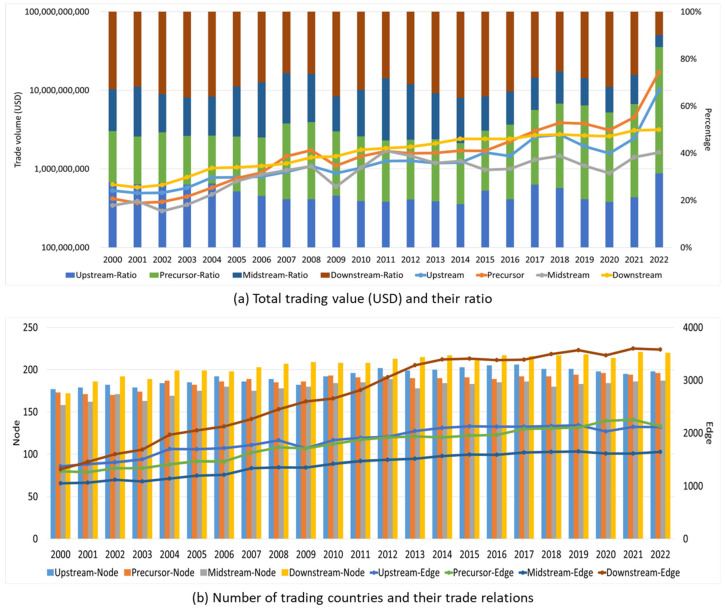
Trade statistics for the upstream, precursor, midstream, and downstream sectors from 2000 to 2021.

Figure 2a shows that the upstream, precursor, midstream, and downstream sectors all experienced substantial growth from 2000 to 2021. Regarding the total trade values, the precursor sector displayed the highest total trade value, followed by the downstream chain, and the midstream chain displayed the lowest value. It is noteworthy to mention that the trade values of the upstream, precursor, midstream, and downstream sectors underwent a fall in 2009, principally ascribed to the consequences of the global financial crisis in 2008. Therefore, this study opted for the years 2000, 2009, and 2021 to undertake a thorough investigation of certain trade network links on a global scale. Regarding the level of countries’ involvement in trade (Figure 2b), it is seen that the upstream and precursor chains involve a larger number of countries compared to the midstream and downstream chains in 2000, with the midstream chain involving the lowest number of countries. It is noteworthy that the downstream industrial chain has gradually come closer to the upstream industrial chain in terms of the number of countries involved. Meanwhile, a substantial number of countries are actively participating in many trade networks, and this tendency is increasing. However, when examining the number of trade relationships, all four types of chains consistently demonstrate a growth trend over time. Among these trading relationships, downstream relationships are the most prevalent, followed by upstream and predecessor relationships, with midstream trade relations being the least prominent. The trade relationships seen in the four main industrial chains show a significant rising trend, with a particular focus on expanding activities in the downstream industry. More precisely, there has been a significant surge in the number of trade links, escalating from 1318 in 2000 to 3600 in 2021.

**Figure 3:**
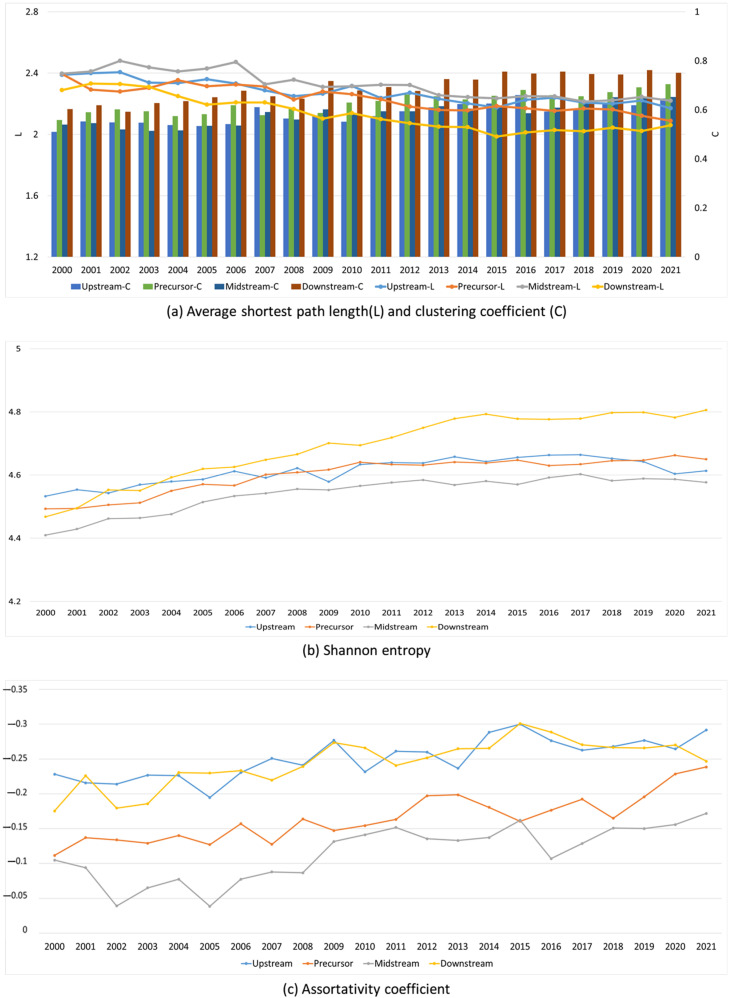
L, C, Shannon entropy, and assortativity coefficient of the upstream, precursor, midstream, and downstream sectors from 2000 to 2021.

Figure 3a illustrates the evolution of the values of L and C. Overall, both L and C exhibit an upward trend. The values of L range between 2 and 2.5, while the values of C consistently exceed 0.6. This suggests that the trade connections between countries in the four different industrial chains are highly interconnected, and this interconnection has become even stronger over time. Furthermore, the downstream sector has the lowest value of L and the highest value of C. This indicates that the countries involved in the downstream industrial chain are the most closely linked among the four industrial chains. In general, the midstream industrial chain has the highest L value and a relatively modest C value, suggesting that it is less interconnected compared to the three other major industrial chains. The Shannon entropy values (Figure 3b) exhibit a small range of 4.4 to 5, indicating a simple and regular network structure among the four distinct industrial chains. Based on the assortativity coefficient (Figure 3c), it can be observed that all industrial chains have a coefficient less than 0. This indicates that all industrial chains exhibit disassortative characteristics, meaning that countries with higher degree values (trade relationships) tend to be connected with countries with smaller degree values. This suggests that the industrial network displays characteristics of multi-core trading countries.

### 4.2. Multilayer Network Structure

**Figure 4:**
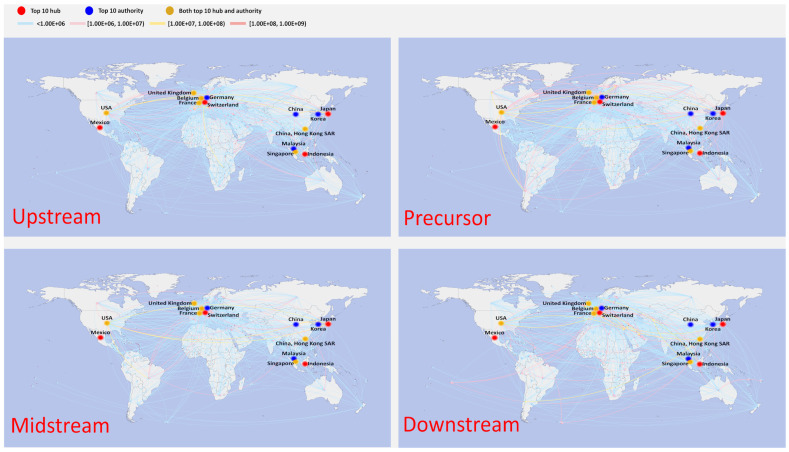
Multilayer structure for traction trade in 2000.

Figure 4 depicts the spatial distribution of trade in 2000. The countries were geographically diverse, with most countries trading in small amounts with each other (34.4% of countries have trade values below USD 100,000, indicated by the light blue line in the graph). This is especially noticeable for less developed regions like Africa and South America. Meanwhile, a small number of countries dominated the trade volume, with the top 20 countries accounting for 84.6% of the total worldwide trade volume. Table 2 displays the ranking of the top 10 countries based on their hub and authority scores. It is evident that most of the countries in the top 10 were situated in East Asia, Europe, and North America. Japan’s exports had the highest hub score, with a significantly higher value compared to other countries. Mexico ranked second, with a value of 0.0938. In terms of import importance ranking (authority), the United States ranked first, followed by Hong Kong SAR of China in second place and Germany in third place. However, the difference in value between the top 10 was not significant.

Table 3 shows the five leading countries in terms of hub and authority scores in the different sections of the industrial chain in 2000. Regarding the upstream sector, the countries primarily involved in import and export activities were mainly concentrated in Europe. The top three exporting countries were the Netherlands, Spain, and Germany, while the top three importing countries were Germany, Belgium, and France. Finland and Chile were the leading exporting countries in the precursor industrial chain, with a very small gap between them. Meanwhile, the United States was also the primary importing country, significantly surpassing Japan, which displayed a value of 0.5216. There was little difference in value between the top two midstream exporters—China and Japan—which accounted for 38.41% of the world’s total. South Korea came in second as an importer (0.5771); however, there was a large gap between it and the United States. As a result of the efforts of Sanyo, Panasonic, Sony, and other Japanese companies to spearhead the completion of research and development and commercialization of the operation, Japan emerged as the leading market for the downstream industrial chain. This country’s exports to 60 countries and total global trade accounted for 40.44 percent of this industry’s value. Mexico ranked second, with a value of 0.0938.

**Figure 5:**
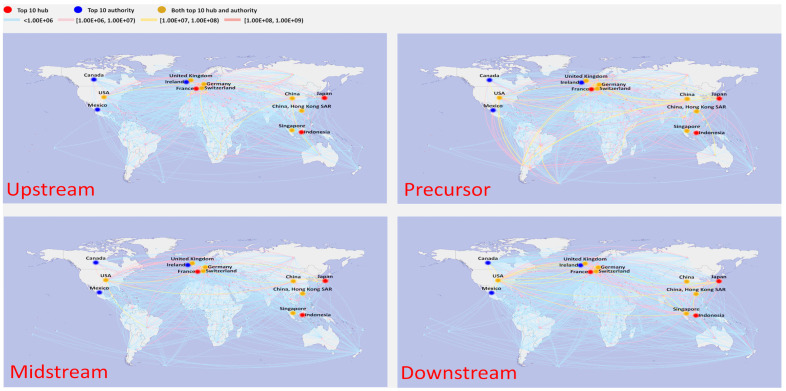
Multilayer structure for traction trade in 2009.

The global trade pattern experienced a fairly substantial shift in 2009 (Figure 5). As Table 2 shows, the United States surpassed Japan (value: 0.2370) to become the leading exporter. Concurrently, most East Asian countries started to surpass their North American and European counterparts as the top-ranking countries; of particular note is the substantial improvement in China’s position. Ireland surpassed the US as the top importer, with Mexico following closely behind with a value of 0.9896. Meanwhile, imports from Europe started to outweigh those from East Asian countries or regions in comparison to 2000. The fast growth of the electric vehicle trade in East Asia led to a complete flip in trade relations, with once-important exporting countries now playing a significant role as importers.

The trade pattern was altered significantly in terms of specific industrial chain, as seen in Table 4, which shows the top five hub and authority countries in 2009. In the upstream sector, China surpassed Germany as the leading mineral importer, while Zambia and Australia supplanted European countries as the leading mineral exporters. China was now the world’s leading exporter for both the precursor and midstream sector, as well as fifth in the world for the downstream sector, providing evidence of the country’s fast ascent in the traction battery industry. While Japan still ranked highest in exports, the United States ranked first in imports of precursors and midstream industrial chains, a role that it played to a lesser degree in its downstream sector. It should be mentioned that Chile continued to play a significant role as an exporter in the precursor sector.

**Figure 6:**
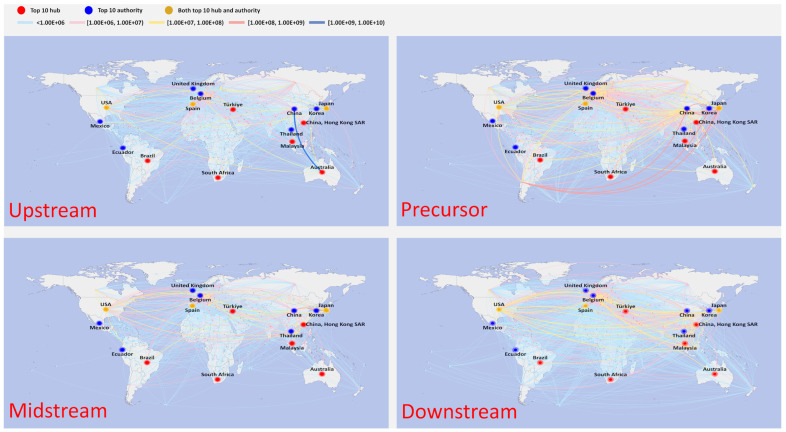
Multilayer structure for traction battery trade in 2021.

Figure 6 depicts the geospatial distribution in 2021. The top 20 ranked countries still accounted for 82.03% of the total global trade value, while the trade value between most countries was minimal, with 21.0% of countries displaying a trade value below USD 100,000. This was particularly the case for Africa, South America, and other comparably poor regions. The top 10 countries based on their hub and authority scores were mainly situated in East Asia, Europe, and North America. China’s rapid progress in traction battery technology has greatly impacted global trade. Compared with 2000 and 2009, there were significant changes in the top 10 countries. The geographic center of trade transitioned from being predominantly centered in Europe to encompassing the global sphere, encompassing regions such as Africa, South America, and others. Notably, Australia emerged as the leading country in exports, while China became the leading country in imports. China’s imports exceeded those of all other countries, and this difference is particularly noticeable.

Table 5 illustrates that the trade volume and trade relations within the particular disperse industrial chains in 2021 were primarily focused within a limited number of countries. In particular, 50.10% of the overall trade volume was made up of Australia’s exports to the rest of the world, while 51.81% was made up of China’s purchases from Australia, Brazil, and Thailand. This is particularly noteworthy given that 47.23% of China’s imports of lithium minerals came from Australia. As a result, China emerged as the top importer and Australia as the top exporter. China’s import value (both at 1.0000) was far higher than other nations’ import values (Brazil at 0.0544 and Belgium at 0.0381, respectively). The main reason for this is that China relies heavily on imports to meet its substantial demand for raw materials in the production of its precursor, midstream, and downstream industries. China dominated midstream trade in terms of exports. It had a significant lead over the second-ranked country, with Chile at 0.5737 and Vietnam at 0.2591. South Korea was the top-ranked country in terms of imports, followed by Japan with a gap. Regarding the downstream sector, there was a minor difference between the trading countries that were top ranked. This suggests that the demand for downstream products is widely distributed and not heavily concentrated in only a few countries.

### 4.3. Versatile Countries in a Global Traction Battery Network

The previous content provides a thorough explanation of the general features of global trade and also highlights the primary countries involved. Hence, this section focuses on the growth of prominent countries, namely Japan and the United States, as well as China and Australia. The objective is to analyze the unique characteristics of trade relations and their evolution trends.

**Figure 7:**
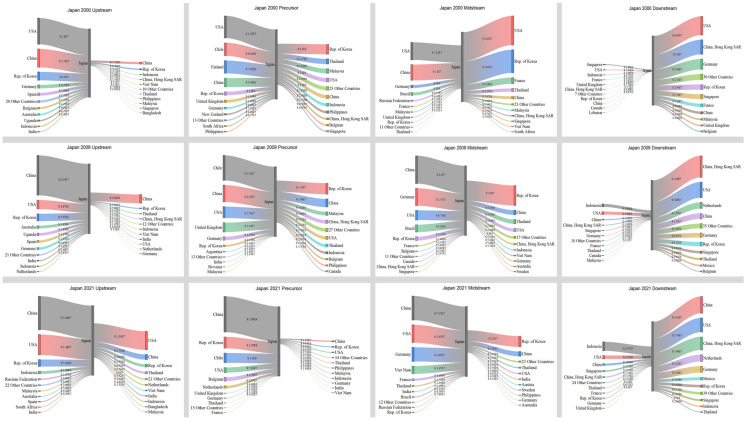
Trade relations and volume of Japan in 2000, 2009, and 2021.

Figure 7 illustrates that in 2000, Japan had a significant need for imports in the upstream, precursor, and midstream sectors. The United States was the primary country involved in Japan’s import trade. Meanwhile, Chile and Finland played significant roles as importers of precursors. However, Japan had a significant export value in the downstream sector, with its primary export destinations being the United States, Hong Kong, China, etc. In 2009, Japan continued to import a significant trade value in the upstream, precursor, and midstream sectors. Notably, China surpassed the United States as Japan’s largest importer in the upstream and midstream sectors, with a trade value of USD 2.15 × 10^7^ and USD 2.5 × 10^7^, respectively. The most significant transformation occurred in the midstream, which mostly relied on imports to meet its demand, resulting in a decline in the value. In 2021, Japan’s trade structure underwent significant changes. The import trade value in the upstream, precursor, and midstream sectors experienced a substantial increase. Additionally, the trade value in the midstream sector doubled, reaching USD 5.19 × 10^7^. Furthermore, China became the largest importer in the precursor trade, with a significant increase in the trade value of USD 3.99 × 10^8^. The trade value of exports declined, and China became the top exporter. Japan predominantly imported raw materials and intermediate goods to meet its substantial need to export battery products. The trade value of battery items displayed a gradual decrease.

**Figure 8:**
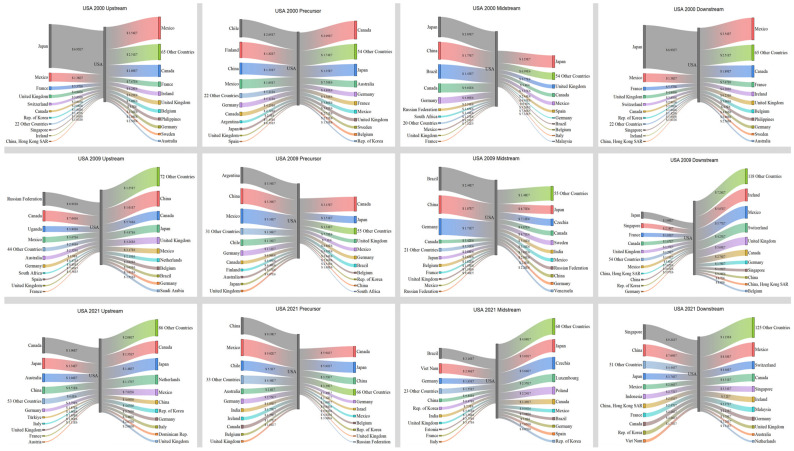
Trade relations and volume of the United States in 2000, 2009, and 2021.

According to Figure 8, in 2000, the United States imported a significant quantity of products. Japan was the primary importer in the upstream, midstream, and downstream sector. Simultaneously, the United States engaged in significant product exports, with its primary exporting partners being countries in North America and Europe. Moreover, the amount of export trade in both the upstream and downstream sectors surpassed that of import trade. In 2009, there was a substantial decline in imports from upstream sources. Concurrently, there was also a fall in its exports. By 2021, there was a notable increase in imports across the four major industrial chains, and a concurrent rise in exports. Additionally, the countries to whom it exported were also experiencing a growing trend. Overall, the United States maintained a highly equitable distribution of imports and exports within its industrial chain.

**Figure 9:**
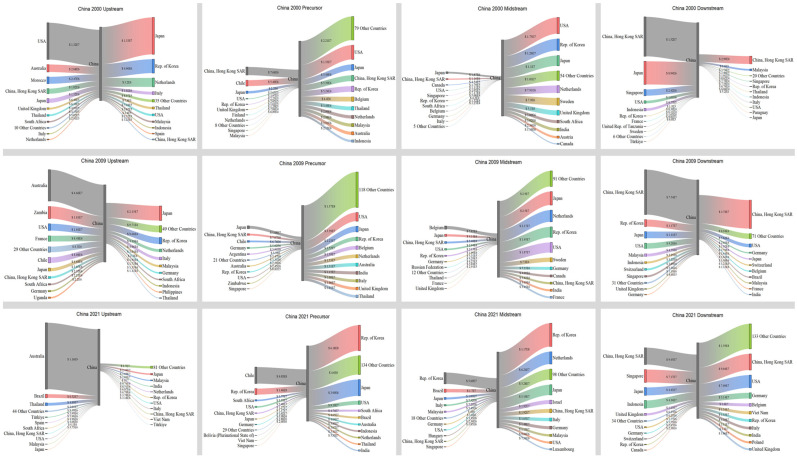
Trade relations and volume of China in 2000, 2009, and 2021.

Figure 9 illustrates that in 2000, China primarily engaged in exporting in the precursor and midstream sectors, with the United States, Japan, and South Korea being the main recipient countries. China mostly fulfilled its trade requirements through imports in the downstream. The largest sources of imports included Hong Kong, China (USD 1.52 × 10^7^), Japan (USD 8.94 × 10^6^), and Singapore (USD 2.42 × 10^6^). In 2009, China’s electric vehicles were in the early stages of their development. The primary factor driving the market was the electric vehicle subsidy program, which resulted in a significant increase in both import and export trade value. Australia emerged as the leading importer in the upstream, with a value of USD 4.66 × 10^7^, compared to USD 2.68 × 10^6^ in 2000. Simultaneously, China’s exports in the downstream experienced a substantial surge, rising from USD 4.47 × 10^6^ in 2000 to USD 7.52 × 10^7^. In 2021, China’s transformation was notably important in three specific areas. Initially, there was a consolidation of upstream imports in terms of both origin and quantity. China’s largest importer remained Australia, but trade value experienced a tremendous increase from USD 4.46 × 10^7^ in 2009 to USD 1.16 × 10^9^ in 2021. The most significant change was observed in China’s exports in the precursor, midstream, and downstream sectors, which showed substantial increases. Specifically, exports in these sectors rose from USD 4.46 × 10^8^, USD 1.42 × 10^8^, and USD 7.52 × 10^7^ in 2009 to USD 1.85 × 10^9^, USD 3.82 × 10^8^, and USD 4.24 × 10^8^ in 2021, respectively. Furthermore, in the downstream, China transitioned from being primarily reliant on imports to becoming a major exporter. China’s exports in this sector (USD 4.24 × 10^8^) surpassed its imports (USD 2.92 × 10^8^). China shifted from being a major exporter in the precursor and midstream chains to becoming the foremost exporter in the precursor, midstream, and downstream chains.

**Figure 10:**
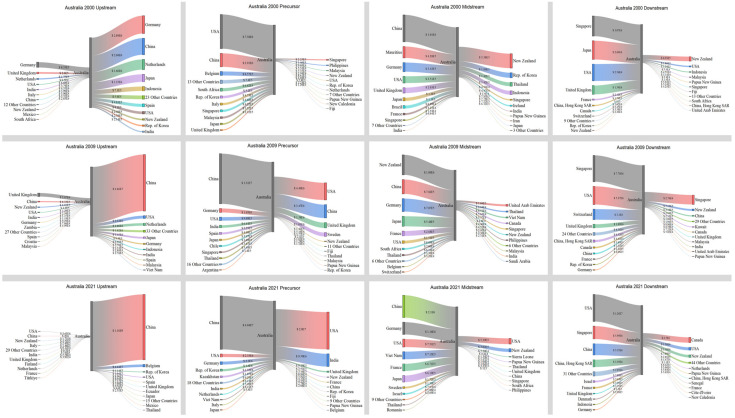
Trade relations and volume of Australia in 2000, 2009, and 2021.

Australia is the primary exporter of minerals and holds the most significant role in upstream exports, particularly compared with Japan, the United States, and China. Figure 10 illustrates that in 2000, the distribution of countries and their export quantities was reasonably even. However, starting from 2009, there was a progressive shift towards China, with a significant increase in trade volume from USD 46.6 million in 2009 to USD 1.16 billion in 2021. In the precursor trade, the United States transitioned from being primarily an importer to also taking on a significant amount of exporting responsibilities, but China took up this position in 2009 and remained the largest importer in 2021. The import quantity increased from USD 7.38 million in 2000 to USD 40.4 million in 2021. The midstream and downstream sectors heavily depend on imports for trade. The midstream sector primarily imports from China and the United States, while the downstream sector mainly imports from Singapore, Japan, the United States, and China.

## 5. Discussion

Given the escalating issue of global climate change, achieving carbon neutrality has become a shared objective among all countries. The objective of achieving carbon neutrality expedites the restructuring of the energy sector, creating significant growth prospects for the electric vehicle industry, and also significantly propelling the rapid advancement of the traction battery industry. Previous research utilized complex network modeling to examine the trade relationships between countries, key trade countries, trading communities, and so on, from the perspective of upstream minerals to downstream traction battery products in global traction battery industrial chains [3,5,17,30]. The findings of this study indicate that the key trading countries and trade relationships of various industrial chain single-layer networks varied significantly. As a result, single-layer complex networks do not sufficiently reveal the inherent correlation characteristics between the trade relations of different industrial chains. This study provides a comprehensive analysis of these trade relationships across the many stages of the worldwide traction battery industry, including the upstream, precursor, midstream, and downstream sectors. The analysis represents the first study conducted from the perspective of a multilayer network for the field of the traction battery industrial chain. Nevertheless, it is evident that despite widespread participation in the traction battery trade, the trade values are highly concentrated in a few countries, with the top 20 contributing over 80% of global trade, which contradicts the collective objective of all countries to promote carbon neutrality. Hence, employing technology-driven poverty alleviation strategies to foster the growth of industrial chains and advance the new energy sector in Africa, South America, and other regions would contribute to the global pursuit of carbon neutrality.

There is a significant dependence between trade relationships in certain countries, and this dependence poses trade risks. Any disruptions in trade due to various factors will result in a risk of interruption in the supply. For instance, China’s rapid development of electric vehicles, fueled by the explosive growth of the traction battery industrial chain, made it the world’s leading producer and consumer by 2021. According to previous research, China is an important country in the lithium trade and consumes almost 50% of the lithium used worldwide. To meet its lithium demands, China mostly imports lithium from Australia and Chile [22]. However, our study highlights that China is the world’s largest importer of upstream mineral raw materials from a single source, and concentrates its trade value in lithium, with more than 90% coming from Australia. Given the current global geopolitical tensions, there is a heightened risk of supply disruptions. The interconnections among various industrial chains mean that any interruption in the trade of essential minerals will significantly impact the entire supply chain, including precursor materials and other minerals necessary for supporting the traction battery industry. Consequently, this will result in the collapse of the traction battery trade on a global scale. Hence, aggressively advocating for the diversification and equalization of crucial trade contacts can mitigate and diminish the dangers inherent in the traction battery trade, supporting the industry’s sustainability and resilience.

## 6. Conclusions

This paper introduces a multilayer trade network of traction batteries, involving the upstream, downstream, midstream, and precursor sectors, based on trade data between all countries around the world from 2000 to 2021. It then uses the multilayer complex network modeling method to conduct a study on the global traction battery field, revealing the features of the spatial distribution of trade relations between countries based on the different sections of the industrial chain and the evolution trend of this distribution. Finally, it analyzes the key trade countries and their development. Research conclusions were drawn from these analyses. (1) The number of trading countries and trade relations involving the four major traction battery industrial chains have shown a notable upward trend over time; in particular, trade relations between downstream countries have grown significantly, rising from 1318 in 2000 to 3600 in 2021. (2) Global trade relations for traction batteries are highly centralized and intimate, with the majority of trade volume concentrated among a small number of trading partners. Several highly ranked countries, with the top 20 countries accounting for over 80% of global trade volume, have had their trade connections strengthen over time. (3) Over time, the geographic center of trade has transitioned from being predominantly centered in Europe, North America, and East Asia to encompassing the global sphere, encompassing regions such as Africa, South America, and Oceania. (4) There have been some changes in the top-ranked trade countries over time. Specifically, Australia replaced Japan as the leading exporter in 2021, while China took over from the United States as the top importer. China’s significant import trade in upstream minerals has played a crucial role in this shift, despite its substantial exports of precursor, midstream, and downstream products. (5) Alterations in trade relationships have influenced the trading patterns of the top-ranked countries to some degree. China stands out as the most prominent example, transitioning from a mineral exporter and downstream importer in 2000 to becoming the world’s foremost trading country by 2021. During this shift, China’s trade pattern transformed into that of an upstream importer and a dominant exporter in the precursor, midstream, and downstream sectors.

There are some limitations to our study. On the one hand, a significant limitation of the UNComtrade data used in this study is the time lag in reporting. Delays of several months or even years can often make data less useful for real-time analysis or decision-making. Rapid technological advancements, market demand, and regulatory changes can quickly make static analyses obsolete, requiring constant updates and recalibrations. On the other hand, the interpretation of results from multilayer network analysis can be more challenging than that of single-layer networks, requiring advanced analytical skills and potentially leading to ambiguous conclusions.

## Figures and Tables

**Table 1:** Data sources and their descriptions.

	Code	Description
Upstream	253090	Lithium. Lithium is commonly utilized in the cathode electrolyte salts of lithium-ion batteries and other related cathode materials.
	260500	Cobalt. The cobalt industry primarily supplies the lithium-ion battery sector. Lithium cobalt (III), a cobalt chemical, is extensively utilized in lithium-ion batteries.
Precursor	282520	Lithium hydroxide. Lithium hydroxide is an important precursor for the preparation of electrolyte materials for traction batteries.
	283321	Manganous sulfate. Manganese sulfate is primarily utilized in lithium-ion batteries for the synthesis of the precursor of the cathode ternary material.
	283324	Nickel sulfate. Nickel sulfate is primarily utilized in lithium-ion batteries for the synthesis of the precursor of the cathode ternary material.
	283329	Cobaltous sulfate. Cobaltous sulfate is primarily utilized in lithium-ion batteries for the synthesis of the precursor of the cathode ternary material.
	283691	Lithium carbonate. Lithium carbonate is a typical building block for lithium-ion battery cathode materials, particularly ternary compositions (LiCoO2, etc.) and those without cobalt (LiFePO4).
Midstream	282590	Lithium cobalt oxides. A crucial component of lithium-ion battery cathode material.
	283529	Iron phosphate. A crucial component of lithium-ion battery cathode material.
	284169	Lithium manganate. A crucial component of lithium-ion battery cathode material.
Downstream	850650	Lithium-ion battery. A final product for traction batteries.

**Table 2:** Top 10 hub countries.

Top 10 Hub Countries						
Rank	2000		2009		2021	
	Country	Value	Country	Value	Country	Value
1	Japan	1.0000	USA	1.0000	Australia	1.0000
2	Mexico	0.0938	Japan	0.2370	Brazil	0.0544
3	France	0.0810	China, Hong Kong SAR	0.2154	Thailand	0.0422
4	USA	0.0792	Singapore	0.1698	Türkiye	0.0083
5	UK	0.0530	China	0.1428	Spain	0.0052
6	Belgium	0.0527	Germany	0.0985	South Africa	0.0051
7	Switzerland	0.0385	France	0.0953	China, Hong Kong SAR	0.0043
8	Indonesia	0.0378	Indonesia	0.0827	USA	0.0041
9	Singapore	0.0289	UK	0.0741	Malaysia	0.0035
10	China, Hong Kong SAR	0.0269	Switzerland	0.0578	Japan	0.0033
**Top 10 Authority Countries**						
1	USA	1.0000	Ireland	1.0000	China	1.0000
2	China, Hong Kong SAR	0.7614	Mexico	0.9896	Belgium	0.0381
3	Germany	0.5652	Switzerland	0.7339	Korea	0.0098
4	Korea	0.2719	UK	0.6761	USA	0.0090
5	Singapore	0.2527	China	0.5055	Spain	0.0008
6	France	0.1772	China, Hong Kong SAR	0.4926	UK	0.0007
7	China	0.1333	Canada	0.4790	Ecuador	0.0006
8	UK	0.0884	Germany	0.3943	Japan	0.0005
9	Malaysia	0.0881	USA	0.2980	Mexico	0.0005
10	Belgium	0.0755	Singapore	0.2670	Thailand	0.0002

**Table 3:** Top 5 hub and authority countries in 2000.

(a) Top 5 Hub Countries in Different Layers								
Rank	Upstream		Precursor		Midstream		Downstream	
	Country	Value	Country	Value	Country	Value	Country	Value
1	Netherlands	1.0000	Finland	1.0000	China	1.0000	Japan	1.0000
2	Spain	0.4778	Chile	0.9620	Japan	0.9273	Mexico	0.0938
3	Germany	0.1461	China	0.7152	Brazil	0.4879	France	0.0810
4	USA	0.1355	USA	0.6079	Canada	0.2774	USA	0.0792
5	Belgium	0.1270	Mexico	0.3644	Germany	0.2392	UK	0.0530
**(b) Top 5 Authority Countries in Different Layers**								
1	Germany	1.0000	USA	1.0000	USA	1.0000	USA	1.0000
2	Belgium	0.6693	Japan	0.5216	Korea	0.5771	China, Hong Kong SAR	0.7614
3	France	0.3348	Germany	0.3556	Japan	0.3033	Germany	0.5652
4	Italy	0.1723	Belgium	0.3234	Netherlands	0.2268	Korea	0.2719
5	Japan	0.1089	Netherlands	0.3202	Sweden	0.1677	Singapore	0.2527

**Table 4:** Top 5 hub and authority countries in 2009.

Top 5 Hub Countries in Different Layers								
Rank	Upstream		Precursor		Midstream		Downstream	
	Country	Value	Country	Value	Country	Value	Country	Value
1	Zambia	1.0000	China	1.0000	China	1.0000	USA	1.0000
2	Australia	0.9265	Germany	0.8069	Germany	0.5999	Japan	0.2370
3	USA	0.2365	Chile	0.6933	Brazil	0.4185	China, Hong Kong SAR	0.2154
4	Spain	0.2317	Argentina	0.3919	Japan	0.1918	Singapore	0.1698
5	France	0.1989	Mexico	0.3411	USA	0.1883	China	0.1428
**Top 5 Authority Countries in Different Layers**								
1	China	1.0000	USA	1.0000	USA	1.0000	Ireland	1.0000
2	South Africa	0.6627	Japan	0.6976	Japan	0.9636	Mexico	0.9896
3	Switzerland	0.2159	Belgium	0.5418	Netherlands	0.7405	Switzerland	0.7339
4	France	0.1328	Korea	0.4810	Korea	0.5840	UK	0.6761
5	Italy	0.1289	Italy	0.4768	Germany	0.2682	China	0.5055

**Table 5:** Top 5 hub and authority countries in 2021.

Top 5 Hub Countries in Different Layers								
Rank	Upstream		Precursor		Midstream		Downstream	
	Country	Value	Country	Value	Country	Value	Country	Value
1	Australia	1.0000	China	1.0000	China	1.0000	Singapore	1.0000
2	Brazil	0.0544	Chile	0.5737	Viet Nam	0.2591	China	0.8257
3	Thailand	0.0422	Korea	0.1409	USA	0.2076	China, Hong Kong SAR	0.6340
4	Türkiye	0.0083	USA	0.0670	Germany	0.1429	Japan	0.5578
5	Spain	0.0052	Russia	0.0470	Japan	0.1256	Indonesia	0.5397
**Top 5 Authority Countries in Different Layers**								
1	China	1.0000	Korea	1.0000	Korea	1.0000	USA	1.0000
2	Belgium	0.0381	Japan	0.6108	Netherlands	0.5424	China	0.8277
3	Korea	0.0098	China	0.3211	Japan	0.5404	China, Hong Kong SAR	0.6880
4	USA	0.0090	USA	0.1584	USA	0.1752	Germany	0.3002
5	Spain	0.0008	Belgium	0.0648	Israel	0.1539	Netherlands	0.2892

## Data Availability

No new data were created or analyzed in this study. Data sharing is not applicable to this article.

## References

[1] IEA (2023). Global EV Outlook 2023.

[2] Hao H., Xing W., Wang A., Song H., Han Y., Zhao P., Xie Z., Chen X. (2022). Multi-layer networks research on analyzing supply risk transmission of lithium industry chain. Resour. Policy.

[3] Chen G., Kong R., Wang Y. (2020). Research on the evolution of lithium trade communities based on the complex network. Phys. A Stat. Mech. Its Appl..

[4] Zhao Y., Gao X., An H., Xi X., Sun Q., Jiang M. (2020). The effect of the mined cobalt trade dependence Network’s structure on trade price. Resour. Policy.

[5] Sun X., Hao H., Liu Z., Zhao F., Song J. (2019). Tracing global cobalt flow: 1995–2015. Resour. Conserv. Recycl..

[6] Shao L., Kou W., Zhang H. (2022). The evolution of the global cobalt and lithium trade pattern and the impacts of the low-cobalt technology of lithium batteries based on multiplex network. Resour. Policy.

[7] Van den Brink S., Kleijn R., Sprecher B., Tukker A. (2020). Identifying supply risks by mapping the cobalt supply chain. Resour. Conserv. Recycl..

[8] Koenig G.M., Belharouak I., Deng H., Sun Y.-K., Amine K. (2011). Composition-tailored synthesis of gradient transition metal precursor particles for lithium-ion battery cathode materials. Chem. Mater..

[9] Fergus J.W. (2010). Recent developments in cathode materials for lithium ion batteries. J. Power Sources.

[10] Lee S., Manthiram A. (2022). Can cobalt be eliminated from lithium-ion batteries?. ACS Energy Lett..

[11] Bridge G., Faigen E. (2022). Towards the lithium-ion battery production network: Thinking beyond mineral supply chains. Energy Res. Soc. Sci..

[12] Tan J., Keiding J.K. (2024). Mapping the cobalt and lithium supply chains for e-mobility transition: Significance of overseas investments and vertical integration in evaluating mineral supply risks. Resour. Conserv. Recycl..

[13] Gonzales-Calienes G., Kannangara M., Bensebaa F. (2023). Economic and environmental viability of lithium-ion battery recycling—Case study in two Canadian regions with different energy mixes. Batteries.

[14] Kampker A., Heimes H.H., Offermanns C., Frieges M.H., Graaf M., Soldan Cattani N., Späth B. (2023). Cost-benefit analysis of downstream applications for retired electric vehicle batteries. World Electr. Veh. J..

[15] Simon B., Weil M. (2013). Analysis of materials and energy flows of different lithium ion traction batteries. Metall. Res. Technol..

[16] De Domenico M., Solé-Ribalta A., Omodei E., Gómez S., Arenas A. (2015). Ranking in interconnected multilayer networks reveals versatile nodes. Nat. Commun..

[17] Tian X., Geng Y., Sarkis J., Gao C., Sun X., Micic T., Hao H., Wang X. (2021). Features of critical resource trade networks of lithium-ion batteries. Resour. Policy.

[18] Kivelä M., Arenas A., Barthelemy M., Gleeson J.P., Moreno Y., Porter M.A. (2014). Multilayer networks. J. Complex Netw..

[19] Watari T., McLellan B.C., Giurco D., Dominish E., Yamasue E., Nansai K. (2019). Total material requirement for the global energy transition to 2050: A focus on transport and electricity. Resour. Conserv. Recycl..

[20] Li Y., Yang J., Song J. (2017). Design structure model and renewable energy technology for rechargeable battery towards greener and more sustainable electric vehicle. Renew. Sustain. Energy Rev..

[21] Guo X., Zhang J., Tian Q. (2021). Modeling the potential impact of future lithium recycling on lithium demand in China: A dynamic SFA approach. Renew. Sustain. Energy Rev..

[22] Song J., Yan W., Cao H., Song Q., Ding H., Lv Z., Zhang Y., Sun Z. (2019). Material flow analysis on critical raw materials of lithium-ion batteries in China. J. Clean. Prod..

[23] Zhou N., Su H., Wu Q., Hu S., Xu D., Yang D., Cheng J. (2022). China’s lithium supply chain: Security dynamics and policy countermeasures. Resour. Policy.

[24] El Hadri H., Mirza D., Rabaud I. (2019). Natural disasters and countries’ exports: New insights from a new (and an old) database. World Econ..

[25] Sturla-Zerene G., Figueroa E., Sturla M. (2020). Reducing GHG global emissions from copper refining and sea shipping of Chile’s mining exports: A world win-win policy. Resour. Policy.

[26] Kim T.-Y., Gould T., Bennet S., Briens F., Dasgupta A., Gonzales P., Gouy A., Kamiya G., Karpiniski M., Lagelee J. (2021). The Role of Critical Minerals in Clean Energy Transitions.

[27] Khurshid A., Chen Y., Rauf A., Khan K. (2023). Critical metals in uncertainty: How Russia-Ukraine conflict drives their prices?. Resour. Policy.

[28] Tabassum S., Pereira F.S., Fernandes S., Gama J. (2018). Social network analysis: An overview. Wiley Interdiscip. Rev. Data Min. Knowl. Discov..

[29] Pavlopoulos G.A., Wegener A.-L., Schneider R. (2008). A survey of visualization tools for biological network analysis. Biodata Min..

[30] Hu X., Wang C., Zhu X., Yao C., Ghadimi P. (2021). Trade structure and risk transmission in the international automotive Li-ion batteries trade. Resour. Conserv. Recycl..

[31] Gupta V., He B., Sethi S.P. (2015). Contingent sourcing under supply disruption and competition. Int. J. Prod. Res..

[32] Interdonato R., Magnani M., Perna D., Tagarelli A., Vega D. (2020). Multilayer network simplification: Approaches, models and methods. Comput. Sci. Rev..

[33] Boccaletti S., Bianconi G., Criado R., Del Genio C.I., Gómez-Gardenes J., Romance M., Sendina-Nadal I., Wang Z., Zanin M. (2014). The structure and dynamics of multilayer networks. Phys. Rep..

[34] De Domenico M., Solé-Ribalta A., Cozzo E., Kivelä M., Moreno Y., Porter M.A., Gómez S., Arenas A. (2013). Mathematical formulation of multilayer networks. Phys. Rev. X.

[35] Peng P., Claramunt C., Cheng S., Yang Y., Lu F. (2023). A multi-layer modelling approach for mining versatile ports of a global maritime transportation network. Int. J. Digit. Earth.

[36] Danziger M.M., Bonamassa I., Boccaletti S., Havlin S. (2019). Dynamic interdependence and competition in multilayer networks. Nat. Phys..

[37] Song H., Luo G., Ji Z., Bo R., Xue Z., Yan D., Zhang F., Bai K., Liu J., Cheng X. (2022). Highly-integrated, miniaturized, stretchable electronic systems based on stacked multilayer network materials. Sci. Adv..

[38] Danziger M.M., Barabási A.-L. (2022). Recovery coupling in multilayer networks. Nat. Commun..

[39] Aleta A., Moreno Y. (2019). Multilayer networks in a nutshell. Annu. Rev. Condens. Matter Phys..

[40] Muldoon S.F., Bassett D.S. (2016). Network and multilayer network approaches to understanding human brain dynamics. Philos. Sci..

[41] Finn K.R., Silk M.J., Porter M.A., Pinter-Wollman N. (2019). The use of multilayer network analysis in animal behaviour. Anim. Behav..

[42] Gao C., Sun M., Shen B. (2015). Features and evolution of international fossil energy trade relationships: A weighted multilayer network analysis. Appl. Energy.

[43] Du W.-B., Zhou X.-L., Lordan O., Wang Z., Zhao C., Zhu Y.-B. (2016). Analysis of the Chinese Airline Network as multi-layer networks. Transp. Res. Part E Logist. Transp. Rev..

[44] Peng P., Poon J.P., Xie X. (2023). COVID-19 Medical Trade: Multilayer Network Analysis and Network Determinants. Netw. Spat. Econ..

[45] Ashton D.J., Jarrett T.C., Johnson N.F. (2005). Effect of congestion costs on shortest paths through complex networks. Phys. Rev. Lett..

[46] Albert R., Barabási A.-L. (2002). Statistical mechanics of complex networks. Rev. Mod. Phys..

[47] Noldus R., Van Mieghem P. (2015). Assortativity in complex networks. J. Complex Netw..

[48] Kleinberg J.M. (1999). Authoritative sources in a hyperlinked environment. J. ACM.

